# Selective Inhibition of Proofreading Exonucleases: The Central Role in Obesity-Associated Carcinogenesis

**DOI:** 10.3390/cimb48040346

**Published:** 2026-03-26

**Authors:** John J. Byrnes

**Affiliations:** Department of Medicine, Division of Hematology, Sylvester Comprehensive Cancer Center, Miller School of Medicine, University of Miami, 1475 NW 12th Ave, Miami, FL 33136, USA; j.byrnes@miami.edu

**Keywords:** AMP, AMPK, carcinogenesis, DNA polymerase, fidelity, energy regulation, metabolism, mutation, obesity, proofreading exonuclease

## Abstract

Obesity-associated carcinogenesis offers a model to explore the transition from metabolic dysregulation to genomic instability and carcinogenesis. Adenosine 5′-monophosphate-activated protein kinase (AMPK), the principal cellular energy sensor, coordinates adenosine triphosphate (ATP) production with metabolic demand; however, in obesity, AMPK activity is impaired, resulting in reduced ATP, elevated Adenosine Monophosphate (AMP), and cellular energy stress. Deoxyribonucleic Acid (DNA) polymerases ε (Pol ε) and δ (Pol δ) maintain replication fidelity via a 3′→5′ exonuclease proofreading activity that removes misincorporated nucleotides. Elevated AMP directly binds and selectively inhibits the exonucleases, conserving energy at the expense of genomic accuracy. As a result, replication errors escape correction and accumulate, some conferring a selective advantage and driving carcinogenic evolution. Therapeutic and lifestyle interventions that activate AMPK—including weight loss, exercise, metformin, and aspirin—restore ATP production, lower AMP, and relieve inhibition of exonuclease proofreading, thereby preserving genomic integrity and slowing mutation-driven carcinogenesis. This framework reveals two core biological principles: 1. Energy metabolism and DNAreplication fidelity are mechanistically coupled at the DNA polymerase active site. 2. The mutation rate is an adaptive metabolic phenotype, modulated by AMP levels. These concepts redefine the metabolic–genetic interface in carcinogenesis and highlight AMPK activation as a rational target for obesity-associated cancer prevention.

## 1. Introduction

The prevalence of obesity is alarmingly high and continues to increase both in the United States and globally. Concomitantly, the incidences of common cancer types are increasing. The convergence of the obesity epidemic with the increasing incidence of various cancers has provided an opportunity to perform a comparative analysis focused on the link between disordered energy metabolism and mutational carcinogenesis. We sought to understand this relationship because such a link would be a target for preventive intervention. The International Agency for Research on Cancer (IARC) highlights that rising obesity significantly drives future cancer burdens, with projections showing millions more cases and huge economic costs due to treatment and lost productivity, demanding urgent prevention strategies to avert future health crises.

In 2003, Calle et al. demonstrated that higher body weight was correlated with increased mortality rates for many cancer types [1]. A 2014 study from the UK reinforced this finding, showing that a high body mass index (BMI) was linked to 13 different cancer types. Every 5 kg/m^2^ increase in BMI elevated the risk of cancers of the uterus, gallbladder, kidney, liver, colon, cervix, thyroid, ovaries, postmenopausal breast, pancreas, rectum, and esophagus, as well as leukemia, multiple myeloma, and meningioma [2]. Based on more than 1000 epidemiological studies worldwide, the IARC reported an association with being overweight, obesity, and weight gain for at least 13 cancers, primarily epithelial-derived adenocarcinomas, including cancer of the esophagus, cancers of the breast (in postmenopausal women), colon, rectum, endometrium (corpus uterus), gallbladder, gastric cardia, kidney (renal cell), liver, ovary, pancreas, and thyroid, as well as meningioma, and multiple myeloma. The strongest association was observed between BMI and endometrial cancer (EC), with a relative risk (RR) of approximately 1.5 for a BMI of 25.0–29.9 kg/m^2^, 2.5 for a BMI of 30.0–34.9 kg/m^2^, 4.5 for a BMI of 35.0–39.9 kg/m^2^, and 7.1 for a BMI > 40.0 kg/m^2^ [3].

The relationship between obesity and cancer begins early in life. A BMI above the normal limit (24.99 kg/m^2^) in early adulthood, age 20, was found to predict an increased risk of colorectal cancer (CRC) and EC [4,5]. Furthermore, it is a continuous process, as the cumulative lifetime excess weight or the weighted years lived overweight or with obesity exhibited an even more robust RR relationship with CRC than a one-time measurement of BMI [6], indicating that the risk factor for obesity-associated cancers is an entity that accumulates, e.g., mutations. The risk of developing obesity-related cancer has increased in ever-younger cohorts in the USA [7].

A strong association of obesity with insulin resistance has been recognized since the mid-1990s. Weight loss often correlates with increased insulin sensitivity [8]. Meta-analyses and cohort studies confirm the association of insulin resistance and type 2 diabetes mellitus (T2DM) with a similar increased cancer risk [9] (as in the obese).

Increases in the prevalence and severity of a definable disorder related to impaired energy metabolism over 20–30 years correlate with BMI. Similarly, the prevalence of various cancers increases in correlation with BMI. On the basis of this observation, we hypothesized that obesity and cancer are metabolically linked, an idea that warranted further analysis.

## 2. Materials and Methods

This study extends our original biochemical discovery of the first eukaryotic DNA polymerase catalytic core possessing intrinsic proofreading exonuclease activity and its previously unexplained selective inhibition, a phenomenon that permits mutation accumulation in newly synthesized DNA. Here, we advance a mechanistic explanation for this observation by integrating experimental enzymology with metabolic regulation in obesity-associated carcinogenesis. By re-examining and repurposing key experimental findings—both our own and others’—as functional results within a unified mechanistic framework, we identify impaired cellular energy metabolism as a causal regulator of DNA polymerase proofreading fidelity. This work defines a previously unrecognized metabolic control point linking AMP-dependent signaling, DNA replication fidelity, and mutational carcinogenesis, thereby transforming a longstanding biochemical observation into a testable pathogenic mechanism.

### 2.1. Genetic and Metabolic Constraints on Proofreading Fidelity

Pathogenic mutations in the proofreading exonuclease domains of the *POLD* and *POLE* genes, encoding DNA polymerase δ and DNA polymerase ε, respectively, provide direct genetic evidence that loss of proofreading activity is sufficient to drive mutational carcinogenesis. Germline and somatic exonuclease-deficient variants produce ultra-mutated tumor phenotypes with markedly increased cancer incidence, establishing proofreading fidelity as a dominant barrier to malignant transformation.

In parallel, metabolic disorders characterized by impaired AMPK signaling—most notably, Peutz–Jeghers syndrome—demonstrate a strong predisposition to epithelial malignancies, implicating energy dysregulation as an upstream permissive factor in tumorigenesis. When considered together, these genetic and metabolic observations converge on a common vulnerability: the energetic cost of high-fidelity DNA replication. These findings motivated our focused analysis of the metabolic modulation of DNA polymerase proofreading as a unifying mechanism of mutation accumulation.

### 2.2. Approach and Rationale

To define a mechanistic link between metabolism and mutational carcinogenesis, we conducted a targeted, hypothesis-driven analysis across two experimentally grounded domains: DNA replication fidelity and energy metabolism in obesity. Rather than cataloging associations, our analysis was explicitly constrained to causal mechanisms capable of modulating mutation formation during DNA synthesis.

Published biochemical, genetic, and metabolic data were interrogated as functional evidence, emphasizing the energetic requirements of nucleotide selection, proofreading exonuclease activity, and mismatch correction. Expertise in DNA polymerase enzymology, cancer genetics, and metabolic regulation was applied to integrate these data into a coherent mechanistic model. This synthesis enabled identification of AMP-mediated regulation of proofreading activity as a metabolic checkpoint governing the trade-off between replication fidelity and cellular survival under energetic stress.

### 2.3. Analysis and Synthesis

Through this process, significant associations and correlations between metabolic, mutational, and carcinogenic factors emerged. Features that regulate both energy metabolism in obesity and mutational carcinogenesis were identified, leading to the synthesis of a tenable mechanism underlying the metabolic linkage between them.

## 3. Results

### 3.1. The Hallmarks of Cancer

#### 3.1.1. The Hallmarks of Cancer and Genomic Instability

In 2000, Hanahan and Weinberg defined six hallmark biological capabilities acquired during the multistep development of human cancers: sustaining proliferative signaling, evading growth suppressors, resisting cell death, enabling replicative immortality, inducing angiogenesis, and activation and metastasis [10].

This framework was expanded in 2011 to emphasize genomic instability as a fundamental enabling characteristic that underlies the acquisition of these hallmarks by generating the genetic diversity required for tumor evolution. Genomic instability is now recognized as a defining feature of cancer, contributing directly to intratumoral heterogeneity, disease progression, and therapeutic resistance. At that time, two additional hallmark capabilities were introduced: reprogramming of energy metabolism and evasion of immune destruction [11].

In 2022, Hanahan further refined the model by identifying phenotypic plasticity and disrupted differentiation as discrete hallmark capabilities, and by also recognizing non-mutational epigenetic reprogramming and polymorphic microbiomes as they drive the accumulation of oncogenic mutations that permit the emergence and maintenance of hallmark traits [12].

Accordingly, understanding carcinogenesis requires understanding relevant genomic instability, including an examination of the mechanisms that normally preserve genomic stability, starting with the fundamentals of eukaryotic DNA replication and fidelity preservation.

#### 3.1.2. DNA Replication Errors and Genomic Instability in Carcinogenesis

**Figure 1:**
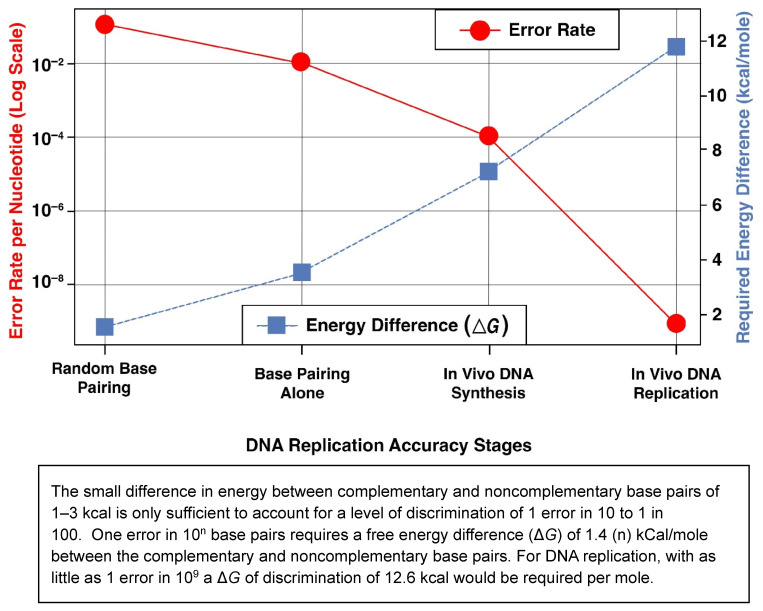
Error rate vs. required energy difference in DNA replication.

Two principal models have been proposed to explain the origin of carcinogenic mutations not of obvious extraneous cause and responsible for genomic instability: the mutator DNA polymerase hypothesis and the stochastic DNA replication error theory. Loeb and colleagues posited that errors introduced during DNA synthesis by cellular polymerases are causally related to malignant transformation. They also emphasized the integral relationship between DNA replication fidelity, genomic stability, and energy cost, noting that higher fidelity demands progressively more energy (Figure 1) [13].

The mutator phenotype hypothesis of oncogenesis was expanded to include defective base excision and mismatch repair, spontaneous deamination of cytosine to uracil, alterations in deoxynucleotide triphosphate pools, and incorporation of ribonucleotides into DNA as sources of mutations [14]. Evidence supporting the mutator phenotype hypothesis came from next-generation sequencing, demonstrating that each tumor is unique, containing tens to hundreds of thousands of mutations, which primarily are misincorporated base substitutions and small base insertions or deletions causing frameshift changes [15]. However, the way most of these mutations occur and the identity of the mutator DNA polymerase have remained elusive.

Vogelstein and collaborators likewise underscored the central role of DNA replication errors in carcinogenesis, proposing a model of malignant transformation being driven by a series of mutations that confer a selective growth advantage to cells [16]. Next-generation sequencing revealed that, while numerous somatic mutations are found in the majority of tumors, most do not enhance the selective growth advantage of cells. The terms “driver” and “passenger” were coined to distinguish between mutations that actively promote carcinogenesis and the numerous others that do not. A mathematical analysis of the evolution of somatic mutations in cancers revealed that the number of mutations in self-renewing tissues correlated with the patient’s age at diagnosis, and indicated that half or more of the somatic mutations are “passenger” mutations, occurring before the onset of neoplasia. Furthermore, many mutations in human cancer were attributable to random errors occurring during somatic cell DNA replication, accumulating as the individual ages. In fact, most human cancers were caused by two to eight sequential driver gene mutations that develop stochastically, accumulating over 20 to 30 years [17]. Further study indicated that the lifetime risk of different cancer types strongly correlates (0.81) with the number of divisions of normal self-renewing cells. Only one-third of the variation in cancer risk among tissues was attributable to environmental or inherited predisposition. Most cases were due to random mutations arising during DNA replication in noncancerous stem cells [18].

Cancer genome sequencing and epidemiological data also indicated that DNA replication errors account for two-thirds of the mutations in human cancers. Approximately 66% of cancer mutations result from DNA replication copying errors, 29% can be attributed to lifestyle or environmental factors, while the remaining 5% are inherited [19]. In common solid tumors, such as those of the colon, breast, brain, pancreas, or endometrium, an average of 33 to 66 genes harbored subtle somatic mutations. Approximately 95% of these mutations were single-base substitutions, with the remainder being deletions or insertions of one or a few bases [20]. Accordingly, an examination of the process of DNA replication and the mechanisms by which the high fidelity of base incorporation is accomplished was incorporated into the analysis.

#### 3.1.3. DNA Replication and Fidelity in Eukaryotes

Eukaryotic genomic DNA replication is primarily carried out by DNA polymerase δ (Pol δ) and DNA polymerase ε (Pol ε). Both enzymes possess intrinsic 3′→5′ exonuclease proofreading activity, which immediately removes misincorporated nucleotides, allowing DNA polymerase to insert the correct nucleotide, and thereby increases replication fidelity by preventing the propagation of replication errors.

The first eukaryotic DNA polymerase exhibiting proofreading activity was identified in 1976 and designated DNA polymerase δ (Pol δ) [21]. This polymerase was recognized as the founding member of a previously uncharacterized class of eukaryotic DNA polymerases, and its 3′→5′ exonuclease function was determined to reside within its 122 kDa catalytic subunit [22]. In 1981, a second proofreading-competent eukaryotic DNA polymerase was described [23]. Initially considered related to Pol δ, this enzyme was subsequently shown to interact with proliferating cell nuclear antigen (PCNA) [24], a sliding-clamp protein essential for highly processive DNA synthesis across extended genomic regions [25].

Further biochemical studies revealed two functional forms of Pol δ: a PCNA-dependent and a PCNA-independent form. In 1989, a highly processive DNA polymerase with 3′→5′ exonuclease activity and independence from PCNA was purified and characterized. This enzyme, which also contains a 122 kDa catalytic core [26], was designated DNA polymerase ε (Pol ε) [27]. As nomenclature evolved, PCNA-dependent polymerases were classified as Pol δ, whereas PCNA-independent polymerases were reclassified as Pol ε [28]. This reorganization subsequently created terminological confusion: the originally described Pol δ, along with the PCNA-dependent form of Pol δ, were concurrently isolated and demonstrated to be structurally and immunologically distinct. Importantly, the originally described enzyme was confirmed to be highly processive and PCNA-independent; nevertheless, in accordance with the revised nomenclature, it was retroactively reassigned as Pol ε, an important distinction for subsequent discussion [29].

Both Pol δ and Pol ε were cloned in the early 1990s, firmly establishing them as the principal replicative polymerases responsible for the bulk of nuclear DNA synthesis in eukaryotic cells [30,31]. A prevalent model has Pol ε synthesizing the leading strand in a continuous manner and Pol δ synthesizing the lagging strand in a discontinuous manner.

##### DNA Polymerase Replication and Stepwise Fidelity Improvements

**Figure 2:**
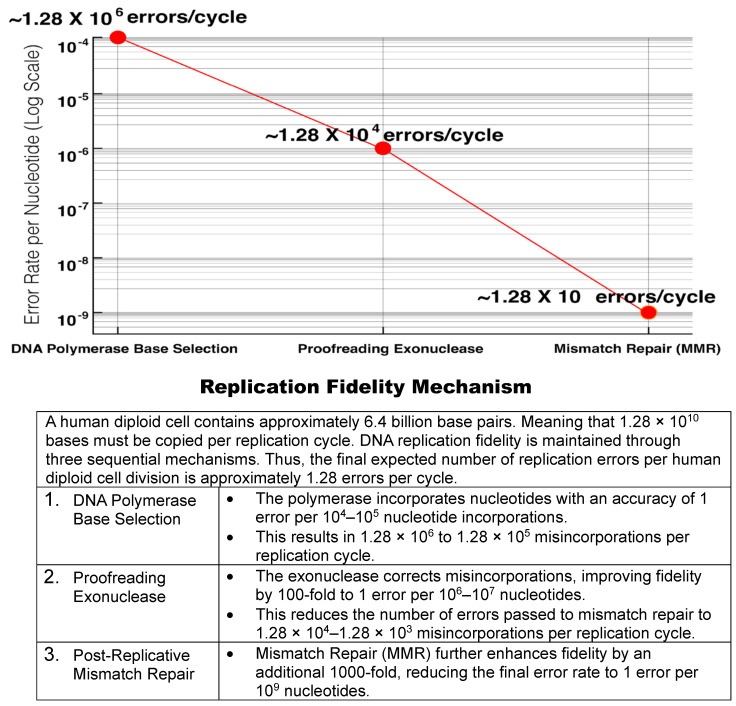
Stepwise reduction of DNA replication errors.

During each cell replication cycle, a human diploid cell genome of 6.4 billion base pairs, or 1.28 × 10^10^ nucleotides, must be copied. The high intrinsic fidelity of the DNA polymerases results in an estimated error rate of more than 1 in 10^5^ nucleotide incorporations. However, this error rate results in roughly 100,000 base misincorporations when copying 6.4 billion base pairs. These misincorporations by the DNA polymerase would be the source of the carcinogenic mutations of the Mutator Hypothesis of Dr. Loeb and the Stochastic Error Theory of Dr. Vogelstein. Eventually, less than 10 errors per cell is arrived at by stepwise reduction of the errors (Figure 2) [32].

#### 3.1.4. Inhibition of Proofreading Activity Is Mutagenic

Selective inhibition of the exonuclease activity of DNA polymerases by nucleotide 5′monophosphates, especially adenosine 5′-monophosphate (AMP), was reported in 1977 as an extension of the description of the first eukaryotic DNA polymerase with proofreading exonuclease activity (Pol δ, reclassified as Pol ε). The addition of AMP to the DNA polymerase reaction selectively inhibited exonuclease activity and decreased fidelity but left polymerase activity unaffected [33]. The half-maximum inhibiting concentration (IC50) of AMP to selectively inhibit the proofreading exonuclease was approximately 150 μM [33].

It is noteworthy that essentially identical findings that 5′ AMP and other 5′ nucleoside monophosphates also selectively inhibit the proofreading exonuclease of *Escherichia coli* DNA polymerase 1 were obtained/demonstrated [34]. The selective inhibition is caused by AMP binding to the product site of the exonuclease [34]. A reason for the functional disassociation of the exonuclease proofreading activity from the DNA polymerase activity induced by AMP, a prevalent metabolite, has remained curiously unexplained.

The effect of selective exonuclease inhibition on eukaryotic DNA polymerase fidelity was examined via the reversion of bacteriophage *M13mp2* mutants. The accuracy of DNA polymerase—referred to as δ II—was high, as it produced fewer than one single-base substitution error for every 10^6^ nucleotides polymerized. Upon AMP-mediated inhibition of this exonuclease activity, overall fidelity decreased, indicating that the exonuclease improved fidelity by as much as 100-fold [35]. The polymerase was later identified as Pol ε. A subsequent study using a forward-mutation assay combined with DNA sequencing provided further insight into the function of the exonuclease. While Pol ε was highly accurate, base substitution and frameshift error rates increased significantly upon the selective inhibition of its exonuclease activity [36]. Since base substitution and frameshift errors are the mutations most responsible for the formation of oncogenes and inactivation of tumor suppressors, a hypothesis was formulated that inhibition of the proofreading exonucleases would be carcinogenic.

#### 3.1.5. Inhibition of Proofreading Activity Is Carcinogenic

Evidence that selective inhibition of proofreading exonuclease activity is carcinogenic was first obtained in mouse experiments. Mice carrying a point mutation (D400A) in the proofreading domain of the Pol δ gene (*POLD*), which selectively disrupted exonuclease proofreading activity while preserving polymerase activity, were used as an experimental model. Homozygous D400A mice began developing cancer as early as 2 months of age, with 94% of these mice developing cancer by 18 months. In contrast, only 3–4% of heterozygous or wild-type mice developed cancer. Among the 66 tumors identified in 49 homozygous mice, 40 were epithelial in origin [37].

Similarly, mice with a mutation in *POLE*, resulting in a Pol ε exonuclease-deficient enzyme with preserved polymerase activity, were developed. Homozygous exonuclease-deficient mice progressively died of cancer between 9 and 24 months of age, with a median survival of 16 months, whereas heterozygotes were indistinguishable from wild-type mice in terms of median survival (median survival = 25 months; *p* > 0.05). The most common tumors observed were intestinal adenomas and adenocarcinomas, with some animals presenting multiple gastrointestinal tumors [38].

Selective inhibition of proofreading exonuclease activity is carcinogenic in humans. Natural knockout mutations in the exonuclease domains of *POLD* and especially *POLE* are strongly linked to the development of cancer. In 2012, the first large-scale cancer genome sequencing study identified tumorigenesis-related *POLE* mutations. Using whole-exome sequencing (WES) and whole-genome sequencing (WGS) of 276 colorectal cancer (CRC) tumors, researchers revealed a subset (16%) of tumors that were hyper-mutated (mutation burden ≥10 mut/Mb) and presented microsatellite instability (MSI). However, another smaller subset (3%) of tumors was classified as ultra-mutated, with a mutation burden exceeding 100 mut/Mb, and *POLE* mutations in these tumors were clustered in the exonuclease domain [39]. Similarly, in 2013, analysis of 373 endometrial carcinomas (ECs) from The Cancer Genome Atlas (TCGA) revealed *POLE* exonuclease-domain mutations in 7% of tumors, with mutation burdens exceeding 100 mut/Mb [40]. These ultra-mutated tumors presented high rates of base substitutions made by the DNA polymerases, demonstrating and underscoring the important role of the exonucleases in preventing carcinogenesis.

Further studies, including a 2013 study by Church et al. in which the exonuclease domains of *POLE* (residues 268–471) and *POLD* (residues 304–517) were screened in 173 EC cases, identified 14 nonsynonymous variants. Thirteen of these nonsynonymous variants were found in *POLE*, and one was found in *POLD*. These variants were linked to the incidence of cancer, with *POLE* mutations predicted to strongly impair proofreading, leading to hyper-mutated tumors [41]. WGS by Palles et al. revealed germline heterozygous mutations in the exonuclease domains of *POLE* and *POLD* in several patients with multiple adenomas and CRC but not in controls. The *POLE* p. Leu424Val and *POLD* p.Ser478Asn variants were identified as susceptibility markers with high penetrance. The *POLD* variant was also associated with an increased risk of EC. These mutations were mapped to equivalent sites in the proofreading exonuclease domains of *POLE* and *POLD* and were predicted to impair the correction of mis-paired bases during DNA replication [42].

A comprehensive review by Rayner et al. in 2016 further established the link between germline *POLE* and *POLD* exonuclease mutations and cancer [43]. Germline mutations in these exonuclease domains were present in 0.5–2% of patients with intestinal polyposis and familial CRC, with *POLE* L424V being the most common deleterious germline variant. This variant was not only associated with CRC but also predisposed patients to EC and increased the risk of other cancers, including breast, stomach, and ovarian carcinomas, as well as brain tumors, duodenal adenomas, and carcinomas. Somatic *POLE* exonuclease-domain mutations were reported in 1–2% of CRC tumors and 7–12% of EC tumors, as well as ultra-mutated tumors of the brain, pancreas, ovary, breast, stomach, and uterus [43].

A 2019 study queried 10,967 cases in the cBioPortal database, and reported 92 *POLE* exonuclease-domain mutations in hyper-mutated tumors [44]. These cases occurred in several cancer types, including EC (9.7%), CRC (2.2%), as well as stomach, adrenocortical, and pancreatic cancers (each with an incidence of 1–2% or lower). Prostate, renal, bladder, and head and neck cancers, cervical squamous cell carcinoma, and melanoma also harbored *POLE* mutations, albeit at lower frequencies. The prevalence of *POLD* exonuclease-domain mutations followed a similar trend [45]. These cancers were primarily epithelial in origin, like the cancer profile associated with obesity, with endometrial (EC) and CRC being especially prominent. Many similar reports followed, and exonuclease-domain mutations are established as a pathological and diagnostic cause of cancer.

Both somatic and germline mutations in the proofreading exonuclease domains of *POLD* and *POLE* demonstrate errors made by the DNA polymerase as a source of oncogenic mutations and genomic instability. They underscore the role of the proofreading exonuclease activity in the prevention of cancers. The hypothesis that inhibition of proofreading exonuclease activity promotes DNA replication-associated carcinogenesis has been confirmed.

### 3.2. Energy Metabolism in Obesity and Associated Disorders

#### 3.2.1. The Hallmarks of Metabolic Syndrome

Metabolic syndrome (MS) was initially recognized as a cluster of interrelated hallmarks that include insulin resistance, obesity, dyslipidemia, and hypertension, contributing to an increased risk of T2DM and cardiovascular disease [46]. Ruderman and Prentki (2004) [47] observed that MS typically occurs in obese patients and is strongly associated with reduced or impaired activation of AMP-activated protein kinase (AMPK), a lack of physical activity, sedentary behavior, overnutrition, and a lack of exercise, all of which contribute to the development of related metabolic disorders. They further noted that multiple interventions known to activate AMPK—including exercise, leptin, adiponectin, metformin, 5-aminoimidazole-4-carboxamide riboside (AICAR), and thiazolidinediones—have been shown to improve insulin resistance and associated metabolic abnormalities in both animal models and humans. On this basis, they proposed impaired AMPK activity as both a central pathogenic factor and a promising therapeutic target in these disorders [47].

#### 3.2.2. Obesity and Associated Disorders Are Characterized by Impaired Energy Metabolism

Obesity and insulin resistance have been extensively linked to impaired energy metabolism, including mitochondrial dysfunction. For example, obese and insulin-resistant individuals exhibit reduced mitochondrial oxidative capacity and defective lipid metabolism compared to healthy, lean controls [8]. Microarray studies have further elucidated the nature of this mitochondrial dysfunction, demonstrating that genes involved in oxidative metabolism-related genes are downregulated in insulin-resistant subjects compared to healthy controls [48,49]. In addition, the expression of genes involved in fatty acid oxidation is significantly reduced in insulin-resistant individuals. Oxidative metabolism is further compromised by decreased expression of multiple genes involved in glycolysis and the tricarboxylic acid cycle. Components of the mitochondrial respiratory chain are also downregulated, including subunits of ATP synthase [49]. Consistent with these transcriptional changes, noninvasive assessment of mitochondrial ATP synthesis rates in young, lean, and sedentary insulin-resistant individuals reveals a 30% reduction compared with insulin-sensitive controls [50].

#### 3.2.3. Impaired Energy Metabolism in Obesity: The Central Role of AMPK

Ruderman and colleagues later expanded the defining hallmarks of metabolic syndrome as “insulin resistance and hyperinsulinemia, central adiposity, dyslipidemia, and predisposition to type 2 diabetes, atherosclerotic cardiovascular disease, hypertension, and certain cancers” [51]. In this updated framework, particular emphasis was again placed on the hypothesis that impaired AMPK activity represents a key pathogenic mechanism, and thus is an important target in prevention and therapy. Consistent with this expanded disease spectrum, Esposito et al. (2012) [52], in an analysis of 38,940 cancer cases, determined that metabolic syndrome (MS) is associated with an increased risk of several primarily epithelial-derived malignancies—liver cancer (RR 1.43, *p* < 0.0001), colorectal cancer (1.25, *p* < 0.001), and bladder cancer (1.10, *p* = 0.013) in men. In women, MS was associated with endometrial cancer (1.61, *p* = 0.001), pancreatic cancer (1.58, *p* < 0.0001), postmenopausal breast cancer (1.56, *p* = 0.017), rectal cancer (1.52, *p* = 0.005), and colorectal cancer (1.34, *p* = 0.006) [52]. As impaired AMPK activity is proposed as a key pathogenic mechanism in these primarily epithelial cancers, it is necessary to consider the role of AMPK in the production of energy to understand the relation to mutational carcinogenesis.

#### 3.2.4. AMP Regulates Energy Production and Consumption

Adenosine triphosphate (ATP) serves as the primary energy currency in cells, driving a wide array of metabolic processes, including DNA replication. Daniel Atkinson proposed that adenine nucleotides regulate branch points between anabolism and catabolism based on the observations of energy-producing metabolic enzymes that directly monitor cellular energy status—such as muscle phosphorylase, fructose bisphosphatase, and phosphofructokinase—which were allosterically regulated by adenine nucleotides, with AMP and ATP acting in a reciprocal fashion. This concept was formulated as the Energy Charge Hypothesis [53], wherein the energy charge was defined as:

$$\mathrm{energy}\;\mathrm{charge}=\frac{\left(ATP\right)+\frac{1}{2}(ADP)}{\left(ATP\right)+\left(ADP\right)+(AMP)}$$

Hans Krebs pointed out that the cellular AMP concentrations fluctuate to a much greater extent than those of ATP or ADP. Normally, cells have a much higher concentration of ATP (5–10 mM) than that of AMP (<0.1 mM). For example, if ATP is consumed such that its concentration drops by 10%, from 5 mM to 4.5 mM, the AMP concentration that was previously 0.1 mM would increase to approximately 0.6 mM, a 600% increase. Although ATP is the primary metabolite that transports high-energy phosphates during metabolic processes via transitioning to ADP or AMP, Krebs concluded that “the absolute concentration of AMP is a much more sensitive controlling agent than the absolute concentration of ATP” [54]. This concept was further established by the discovery of AMP-activated protein kinase (AMPK). Acetyl-CoA carboxylase kinase was found to be stimulated by AMP, and it was suggested that this mechanism might inhibit fatty acid synthesis in response to falling energy charge [55], and HMG-CoA reductase kinase was also found to be stimulated by AMP [56]. In 1987, Carling et al. reported that a single protein kinase could account for both observations [57]. When it became evident that the kinase had multiple physiological substrates, it was named “AMP-activated protein kinase” (AMPK), after its allosteric activator, AMP [58]. AMPK is activated in an ultra-sensitive manner by cellular stresses that deplete ATP and, consequently, elevate AMP concentrations. AMP binding to AMPK was found to have three regulating effects: (i) a conformational change that activates and makes AMPK a good substrate for phosphorylation at Tyr 172 of the catalytic subunit by another kinase, namely, LKB1; (ii) phosphorylation of this tyrosine residue by LKB1 potentiates further AMP-induced activation of AMPK (10-fold); and (iii) inhibition of Tyr 172 dephosphorylation. These three mechanisms act synergistically and make the system sensitive to small increases in AMP [59]. AMPK activation switches on catabolic ATP synthesis pathways while suppressing many ATP-consuming, anabolic processes. As a result, ATP concentration is increased, while AMP concentration is reduced. The half-maximal effective concentration (EC50) for AMP activating AMPK under physiological conditions was determined to be 196 μM [60], implying that this concentration of AMP represents an energy-deficient state.

The discovery of AMPK reinforced the Energy Charge Hypothesis, with cellular AMP concentration coordinating energy production and consumption. “Any system that monitors cellular energy status should respond to AMP concentrations” [61]. A relatively high AMP occurs when the energy charge is low and should be conserved and ATP production increased; conversely, cellular AMP is low when the energy charge—ATP + ½ ADP—content is high.

In mammals, AMPK is a heterotrimeric complex with an α catalytic subunit as well as β and γ regulatory subunits. Each subunit is encoded by two or three genes (α1, α2, β1, β2, γ1, γ2, and γ3), and at least 12 heterotrimeric combinations are possible, providing a diversity of regulatory functions [62]. These combinations confer different properties to AMPK, including subcellular localization and signaling functions. Inhibitory regulation of AMPK by nutrients, lipid overload, high-glucose concentrations, glycogen, various amino acids, hormones, cytokines, and especially inflammatory signals has been shown to be relevant to disorders of impaired energy metabolism [63].

In summary, AMPK is the major regulator of cellular energy production and consumption at multiple and diverse levels in the control of whole-body homeostasis and the promotion of cell survival under conditions of low cellular energy, with the number of reported targets for AMPK currently more than one hundred; these include the regulation of a wide array of other physiological events, such as cellular growth and proliferation, mitochondrial function, and biogenesis, and factors that have been linked to insulin resistance, such as inflammation, oxidative stress, and autophagy [64].

#### 3.2.5. Impaired AMPK Activity Is Linked with Carcinogenesis

Impaired AMPK activity is a key pathogenic mechanism in certain cancers. The Peutz–Jeghers syndrome (PJS) links impaired AMPK-regulated energy metabolism with (epithelial cell) carcinogenesis. The RR of epithelial-derived carcinomas, similar to that of obesity, is 15.2 times greater in patients with familial PJS than in the general population [65]. In 1998, Hemminski et al. [66] and Jenne et al. [67] reported that PJS is caused by inactivating mutations in the serine-threonine kinase LKB1, indicating that LKB1 is a tumor suppressor. In 2003, Hawley et al. [68], Woods et al. [69], and Shaw et al. [70] reported that LKB1 is the upstream kinase required for AMPK activation, thus linking impaired AMPK regulation with carcinogenesis. Hardie noted that “the most significant impact of this discovery was the connection between LKB1, a tumor suppressor involved in cancer, and AMPK, a protein kinase previously recognized for its role in regulating metabolism, particularly in disorders like diabetes” [71]. Thus, LKB1 deficiency impairs AMPK and establishes a mechanistic connection between impaired energy metabolism and epithelial cell carcinogenesis.

#### 3.2.6. AMPK Activation Prevents These Cancers

AMPK activity in the adipose tissue of obese, insulin-resistant individuals before undergoing bariatric surgery was found to be reduced by thirty to fifty percent [72,73]. Weight loss by procedures such as bariatric surgery reverses insulin resistance and the metabolic disorder associated with MS. Notably, bariatric surgery was found to lower the prevalence of solid (obesity-associated) cancers by 70% within five years [74].

Similarly, Hardie hypothesized that “if evidence suggests that the LKB1-AMPK pathway is indeed a tumor-suppressing pathway, then AMPK-activating drugs might be expected to provide protection against the development of cancer” [71]. A 2005 study reported that patients with T2DM receiving metformin, a known AMPK activator, had a significantly reduced incidence of cancer [75]. This finding was subsequently validated in other T2DM patient cohorts, as a meta-analysis indicated an overall cancer RR of 0.61 (95% confidence interval [CI] [0.54–0.70]) in metformin users compared with sulfonylurea users [76].

Hardie also proposed that AMPK activation can explain some of the beneficial effects of salsalate and aspirin. These drugs break down into salicylate, which activates AMPK by binding at an allosteric site, inhibiting the dephosphorylation of AMPK at Thr172 [77]. The cancer-preventive effect of aspirin was first reported after long-term follow-up in large trials designed to evaluate its cardiovascular benefits. Data from 51 trials, including more than 77,000 participants, demonstrated that individuals had a reduced risk of cancer after five years of aspirin administration (hazard ratio [HR]: 0.81, 95% CI: 0.72–0.93) [78].

Obesity and EC are strongly and significantly associated, suggesting that this relationship is suitable for exploring the potential application of AMPK activators for cancer prevention. Several large studies have demonstrated the effectiveness of aspirin in preventing EC, particularly in obese patients [79]. For example, the Australian National EC Study, comprising 1398 cases, revealed a nearly 50% risk reduction (OR = 0.54, 95% CI: 0.38–0.78) in women reporting the use of ≥2 aspirin tablets per week. A meta-analysis of eight previous studies combined with these data suggested that aspirin use was associated with a lower cancer risk, with a pooled risk estimate for obese women (BMI > 30 kg/m^2^) of 0.72 (95% CI: 0.58–0.90). However, no significant association between aspirin use and cancer risk was observed among nonobese women (BMI < 30 kg/m^2^) [80].

Another meta-analysis of 13 observational studies, including 11,323 cases, demonstrated that regular aspirin use was associated with a decreased risk of EC. When patients with the highest frequency of use were compared with nonusers, a risk reduction of 37% (OR = 0.63, 95% CI: 0.45–0.88) was observed. Analysis with respect to BMI revealed an inverse association of EC risk reduction among women with a BMI > 30 kg/m^2^ (case-control: 44% risk reduction, OR = 0.56, 95% CI: 0.33–0.95; cohort: 20% risk reduction, OR = 0.80, 95% CI: 0.60–1.07) [81]. Further analysis revealed a greater risk reduction with increasing aspirin dose and frequency, with long-term use conferring protection from EC mainly in women with obesity [82].

Finally, a subgroup analysis of data from over 7000 women with EC from the Epidemiology of Endometrial Cancer Consortium revealed that the use of aspirin at least once weekly was associated with a risk reduction (OR = 0.86, 95% CI: 0.76–0.98) in overweight and obese women, whereas no effect was observed in women of normal weight [83].

Aspirin has also been shown to reduce the risk of CRC, particularly in obese patients. In the Colorectal Adenoma/Carcinoma Prevention Program (CAPP), individuals with Lynch syndrome were randomly assigned to 600 mg/day aspirin or a placebo. Long-term analysis demonstrated a marked reduction in the incidence of CRC (incidence rate ratio: 0.37) in participants taking aspirin for two or more years compared with those receiving a placebo [84].

A recent meta-analysis confirmed the association of regular aspirin use with a significantly reduced risk of cancers, including CRC (RR = 0.73, 95% CI = 0.69–0.78, 45 studies); squamous cell esophageal cancer (RR = 0.67, 95% CI = 0.57–0.79, 13 studies); adenocarcinoma of the esophagus and gastric cardia (RR = 0.61, 95% CI = 0.49–0.77, 10 studies); stomach cancer (RR = 0.64, 95% CI = 0.51–0.82, 14 studies); hepato-biliary tract cancer (RR = 0.62, 95% CI = 0.44–0.86, five studies); and pancreatic cancer (RR = 0.78, 95% CI = 0.68–0.89, 15 studies). A dose- and time-dependent linear response was observed for CRC, with a significant 10% reduction with 75 mg/day aspirin increasing to 50% with 500 mg/day aspirin. Patients exhibited a time-dependent RR of 4%, 19%, and 29% at 1, 5, and 10 years, respectively [85]. Obesity was not included in the analysis. However, in a complementary cohort study of 107,655 men and women receiving aspirin and followed up for more than 3 decades, aspirin reduced the absolute risk of CRC primarily in individuals with obesity, supporting the thesis that AMPK activation in obesity reduces the risk of the associated carcinogenesis [86].

A consideration of the prominence of epithelial cancer in obesity and associated disorders is warranted. Epithelial tissues are characterized by continuous and highly ordered cell turnover and proliferation, requiring tightly synchronized cycles of DNA replication, mitosis, and differentiation to maintain tissue integrity and barrier functions. Large populations of cells enter the cell cycle in a coordinated fashion in response to physiological cues. As a result, epithelial cells operate near the upper limits of replicative capacity under normal conditions, leaving little tolerance for perturbations in cell-cycle control, nucleotide availability, or energy homeostasis. Their failure to participate could have immediate life-threatening consequences. Consequently, epithelial replication fidelity is intrinsically linked to cellular energy status and to the metabolic pathways that sustain nucleotide pool balance and ATP/AMP homeostasis.

Endometrial epithelial cells are prototypical in this regard. Across the menstrual cycle, the endometrium undergoes repeated rounds of hormonally driven proliferation, differentiation, shedding, and regeneration. During the proliferative phase, endometrial epithelial cells engage in rapid and synchronized DNA synthesis, making them exquisitely dependent on intact energy metabolism and robust nucleotide biosynthesis. This recurrent, large-scale replicative activity renders the endometrium particularly vulnerable to fluctuations in energy charge and mechanisms that coordinate energy availability with DNA replication fidelity.

## 4. Discussion

The hypothesis that selective inhibition of proofreading exonuclease activity leads to carcinogenesis by failing to correct DNA polymerase base substitutions and frameshift errors has been confirmed [37,38,39,40,41,42,43,44,45]. The hypotheses that impaired AMPK-regulated energy production in obesity contributes to carcinogenesis [65,66,67] and, conversely, that AMPK activators can reduce the risk of obesity-associated cancers have also been validated [75,76,77,78,79,80,81,82,83,84,85,86]. These concepts are interconnected through increased AMP, which, under conditions of low cellular energy, inhibits the proofreading exonucleases to conserve energy and promote cell survival [32,36,60,61]. Increased AMP also attempts to increase energy production and decrease consumption; however, AMPK is impaired [46,51]. Therefore, we postulate that AMP-regulated proofreading activity underlies obesity-associated carcinogenesis, and we propose the following mechanism and synthesis.

In obesity, impaired AMPK activity leads to decreased ATP levels [46,51], causing a relatively higher increase in AMP levels, as described by Krebs [54]. Increased AMP inhibits DNA polymerase proofreading exonuclease activity, reducing the correction of misincorporated bases [33,34,35,36], some of which give rise to oncogenic mutations or disrupt tumor suppressor genes [35,36]. Conversely, if AMPK is reactivated, increasing ATP production and decreasing AMP levels allows proofreading activity to resume, thereby reducing the accumulation of carcinogenic mutations and lowering cancer risk over time [75,76,77,78,79,80,81,82,83,84,85,86]. The AMP concentration selectively inhibits DNA replication proofreading activity in accordance with available energy levels, which are reduced in the circumstance of impaired AMPK in obesity and associated disorders.

The selective inhibition of exonuclease-mediated proofreading by AMP differs from inhibition caused by exonuclease-domain mutations. AMP-mediated inhibition is conditional rather than permanent, fluctuating across different cells and timeframes depending on the metabolic conditions and energy availability. In contrast, loss of exonuclease activity due to domain mutations is permanent and pathological. Despite differing mechanisms, both AMP-driven inhibition and exonuclease gene mutations contribute to mutational carcinogenesis in a similar manner by allowing base substitutions and frameshift errors made by the DNA polymerase to remain uncorrected and accumulate within the genome (Table 1).

Regulating the mutation rate in relation to available energy introduces a third function to the DNA polymerase catalytic core, alongside DNA synthesis and exonuclease proofreading [22,33,34]. Thus, selective inhibition of the proofreading activity by AMP establishes both a metabolic and physical link between energy metabolism and mutational carcinogenesis. This metabolic linkage should be a primary target for intervention. Specifically, increasing cellular ATP concentrations to lower AMP levels and restoring proofreading exonuclease activity by activating impaired AMPK represents a viable strategy to enhance DNA replication fidelity and mitigate obesity-associated carcinogenesis. The referenced clinical studies support the carcinogenic-preventive effectiveness of this approach [75,76,77,78,79,80,81,82,83,84,85,86].

This analysis reveals two key insights:

1. The mutation rate is metabolically regulated via energy-sensitive proofreading modulation;
2. This regulation is integrated within the single 122 kDa core of the DNA polymerase complex [22], linking impaired energy metabolism directly to genomic instability.

Together, they indicate that the prevention of mutational oncogenesis requires an integration of the disciplines of energy metabolism and DNA replication enzymology, both in the research laboratory and the clinic. These findings explain the epidemiological connection between obesity and cancer and offer a mechanistic framework for understanding how impaired energy metabolism may promote carcinogenesis. The implications for prevention and therapeutic intervention are considerable, particularly—but not exclusively—for obesity-associated cancers.

Within the Hallmarks of Cancer paradigm [10,11,12], this model positions impaired energy metabolism not merely as an adaptive feature of cancer cells, but as a causal upstream driver of mutational oncogenesis. It explains how the metabolic dysfunction characteristic of obesity can precede and promote tumor initiation by enabling genome instability, thereby accelerating cancer evolution.

Within the Hallmarks of Cancer paradigm, this synthesis includes two “enabling characteristics”:

1. “Genomic Instability and Mutation”: Impaired energy metabolism, increasing AMP-mediated inhibition of DNA polymerase proofreading, accelerates stochastic mutation accumulation and genomic instability.
2. “Dysregulated Cellular Energetics”: Obesity-associated AMPK impairment represents a primary metabolic lesion that precedes and facilitates genomic instability.

And core hallmarks are also influenced indirectly.

1. “Sustaining Proliferative Signaling”: An increased mutational burden raises the probability of activating driver mutations.
2. “Resisting Cell Death”: Energy stress–induced prioritization of survival over fidelity favors short-term viability at the cost of long-term genomic integrity.
3. “Tumor Evolution and Heterogeneity”: A higher mutation rate increases clonal diversity and accelerates evolutionary selection.

This synthesis is also consistent with the second law of thermodynamics: increasing informational order—in this case, the accurate copying of genetic information—necessarily requires the dissipation of free energy. In practical terms, each incremental reduction in mutation frequency requires a disproportionate increase in energy expenditure, producing a nonlinear, approximately logarithmic relationship between replication fidelity and energetic cost, as emphasized by Loeb [13]. Thus, the maintenance of genomic stability is contingent upon adequate energy availability, whereas energy limitation favors a shift towards lower-fidelity DNA replication that conserves ATP at the expense of increased mutational burden. Consequently, DNA replication fidelity emerges as a metabolically regulated parameter rather than a fixed biochemical constant. Under conditions of impaired energy metabolism, such as those observed in obesity and insulin resistance, energetic constraints may selectively relax proofreading and repair mechanisms, thereby increasing mutation rates and promoting genomic instability. This energetic perspective provides a unifying physical and biochemical framework linking cellular metabolism, thermodynamic constraints, and carcinogenic mutation accumulation. At last, there is a metabolically reasonable explanation for the selective inhibition of the proofreading exonucleases by a prevalent regulatory metabolite.

Limitations and corollary questions are evident. Research on energy metabolism has focused on specific tissues, including skeletal muscle, adipose tissue, and liver, with findings frequently extrapolated to carcinogenic epithelial tissues due to the separation of the two disciplines. A central goal of our work is to ensure that these limitations and mechanistic uncertainties are recognized and addressed in future research through integrated, unified studies of energy metabolism with cancer prevention.

The cause of impaired AMPK activity in obesity represents an important corollary question. Proinflammatory cytokines associated with obesity, such as tumor necrosis factor-α (TNF-α), interleukin-6 (IL-6), and others, have been implicated; this remains an area of active investigation and evolving science.

The scientific and pharmacological discovery of AMPK activation is an area of substantial interest, with numerous natural products and several synthetic compounds under consideration for further development. There is intense interest in the pharmacological activation of AMPK as a therapeutic strategy for multiple disease states, including obesity, diabetes, and cancer [87].

## 5. Conclusions

This synthesis encompasses a number of practical implications. Impaired AMPK in obesity provides a mechanistically coherent link between disordered energy metabolism and carcinogenesis. This framework is intuitive, biologically grounded, and supports existing recommendations for weight control, diet quality, and physical activity, potentially improving long-term adherence. Importantly, the predicted reduction in cancer risk operates on an extended time horizon: if carcinogenesis is driven by stochastic DNA replication errors whose frequency is modulated by cellular energy status, then metabolic correction reduces the rate of mutation accumulation rather than reversing existing damage. Consequently, measurable oncological benefit may not be evident for many years.

In contrast, improvements in cardiometabolic health, including insulin sensitivity, hepatic steatosis, glycemic control, and systemic inflammation, often/can occur within weeks to months, and should be emphasized as primary motivators. Cancer reduction should be framed as a long-term consequence of sustained metabolic normalization.

Pharmacological agents, such as GLP-receptor agonists, will likely reduce obesity cancer risk indirectly through sustained weight loss. Since their primary actions are centrally mediated, tissue-specific consequences preclude simple mechanistic predictions.

Finally, continuous AMPK overstimulation may have adverse tissue-specific consequences, underscoring that prevention aims to restore physiological, context-dependent AMPK regulation rather than induce chronic activation. The history of low-dose aspirin in primary prevention provides a useful and relevant analogy: modest long-term benefit can be offset by cumulative adverse effects, underscoring the need for balance and selectivity in preventive strategies.

We hope this synthesis of cellular energy metabolism and its relationship to obesity-associated carcinogenesis, particularly in terms of preventive strategies, will encourage its further development and its application to alleviate the projected rise in cancer cases.

“However, in real life it is survival, not fidelity, that is the ultimate virtue” (Radman) [88].

## Figures and Tables

**Table 1:** Selective inhibition of proofreading exonucleases plays the central role in obesity-associated carcinogenesis.

Normal	Exonuclease-Inactivating Domain Mutation in One of Four DNA Polymerase δ or ε Genes	Obesity-Impaired AMPK Energy Metabolism Causes Increased Cell AMP Levels That Selectively Inhibit the Proofreading Exonucleases
DNA polymerases epsilon and delta (δ and ε) make approximately 100,000 nucleotide misincorporations per cell replication cycle.	DNA polymerases δ and ε make approximately 100,000 nucleotide misincorporations per cell replication cycle.	DNA polymerases δ and ε make approximately 100,000 nucleotide misincorporations per cell replication cycle.
The proofreading exonucleases increase fidelity 100-1000-fold, removing about 99,000 to 99,900 misincorporated nucleotides, allowing for their correction. About 100 to 1000 remain as mutations; some may be a cancer driver.	The mutated DNA polymerases without proofreading exonucleases continue to replicate DNA but are unable to correct misincorporations, leaving them as numerous mutations.	The proofreading exonucleases of all δ and ε enzymes are selectively inhibited in accordance with the obesity-associated increased cell AMP levels, resulting in a greater-than-normal number of mutations left for MMR correction.
Post-replicative mismatch repair (MMR) corrects most of the rest.	MMR is overwhelmed, and many mutations escape correction—some are drivers.	The greater-than-normal number of mutations allows some to escape MMR correction and accumulate in the cell’s genome over many cell replications occurring over time.
An occasional epithelial cancer may occur in late life.	Frequent epithelial ultra-mutated cancers occur starting early in life.	The increased accumulation of mutations, some of which are cancer drivers, is in accordance with the lifetime weighted degree of obesity (AMP level), causing a significant increase in obesity-associated cancer risk, with some cancers occurring earlier in life than usual.

Note: Carcinogenic consequences of human 6.12 × 10^9^ base pairs per genome being replicated by DNA polymerases δ and ε with a nucleotide misincorporation rate of one error in 10^5^ incorporations under different proofreading conditions: normal, impaired proofreading due to exonuclease-domain mutations, and increased cell AMP due to obesity-associated impaired energy metabolism. AMP selectively inhibits the proofreading exonucleases (refs. [32,33]).

## Data Availability

No new data were created or analyzed in this study. Data sharing is not applicable to this article.
